# Association of alcohol consumption with prevalence of fatty liver after adjustment for dietary patterns: Cross-sectional analysis of Japanese middle-aged adults

**DOI:** 10.1016/j.clnu.2019.07.001

**Published:** 2020-05

**Authors:** Ryoko Tajima, Fumiaki Imamura, Takeshi Kimura, Satomi Kobayashi, Katsunori Masuda, Kaoruko Iida

**Affiliations:** a Department of Nutrition and Food Science, Graduate School of Humanities and Sciences, Ochanomizu University, 2-1-1 Otsuka, Bunkyo-ku, Tokyo 112-8610, Japan; b Department of Social and Preventive Epidemiology, School of Public Health, The University of Tokyo, 7-3-1 Hongo, Bunkyo-ku, Tokyo 113-0033, Japan; c Medical Research Council Epidemiology Unit, Institute of Metabolic Science, University of Cambridge School of Clinical Medicine, Cambridge Biomedical Campus, Cambridge, CB2 0QQ, United Kingdom; d Center for Preventive Medicine, St. Luke's International University, 10-1 Akashi-cho, Chuo-ku, Tokyo 104-0044, Japan; e The Institute for Human Life Innovation, Ochanomizu University, 2-1-1 Otsuka, Bunkyo-ku, Tokyo 112-8610, Japan

**Keywords:** Alcohol intake, Dietary patterns, Diet history questionnaire, Fatty liver, BDHQ, brief-type self-administered diet history questionnaire, BMI, body mass index, CI, confidence interval, NAFLD, non-alcoholic fatty liver disease, PR, prevalence ratio, SD, standard deviation, %E, percentage of energy

## Abstract

**Background & aims:**

Moderate alcohol intake is associated with reduced prevalence or incidence of fatty liver. However, whether or not the association is independent of dietary patterns remains unclear. We aimed to evaluate the cross-sectional association of alcohol intake with fatty liver after accounting for dietary patterns and obesity.

**Methods:**

We assessed 4579 adults aged 30–79 years who participated in routine clinical examinations in St. Luke's International Hospital, Japan (January to March, 2015). We assessed their habitual diet using diet-history questionnaire, estimated alcohol intake, and derived dietary pattern variables using factor analysis. Fatty liver was ascertained using ultrasonography. Linear and U-shaped associations of alcohol intake with fatty liver were evaluated using Poisson regression, and a post hoc analysis was conducted after detecting potential outliers for alcohol intake and excluding them using sex-specific statistics (median plus 2 × interquartile range).

**Results:**

Fatty liver was ascertained in 1120 participants (24.5%). Whereas no significant association of alcohol intake with fatty liver was observed when potential outliers of alcohol intake were included (p = 0.25), a significant U-shaped association was observed after excluding the outliers with and without adjustment for dietary patterns (p = 0.003 and 0.02, respectively). The lowest prevalence was estimated when alcohol consumption was approximately 7% of energy, with a prevalence ratio of 0.72 (95% confidence interval = 0.59–0.86) compared to non-drinkers. The association became imprecise and attenuated toward the null after further adjustment for body mass index (p = 0.06).

**Conclusions:**

Alcohol intake showed a U-shaped association with fatty liver prevalence. This association was independent of underlying dietary patterns, while it was sensitive to excessive alcohol intake and obesity status, providing clinical implications for the prevention of fatty liver.

## 1 Introduction

Fatty liver is a major public health concern, and is classified into alcoholic fatty liver disease and non-alcoholic fatty liver disease (NAFLD). Both types of fatty liver can progress to steatohepatitis and further progress to cirrhosis and hepatocellular carcinoma, which increase premature mortality [1]. Excess alcohol intake is a well-known modifiable risk factor that leads to liver diseases including fatty liver [2], [3], [4], [5], [6]. Although the definition of “excess alcohol intake” is inconsistent across countries, NAFLD is generally distinguished from alcoholic fatty liver disease based on alcohol intake <20 or 30 g/day for men and <10 or 20 g for women. However, despite the general consensus that alcohol consumption causes fatty liver, cross-sectional [7], [8], [9], [10], [11], [12], [13], [14], [15] and prospective studies [8], [16], [17], [18], [19] have reported that moderate to heavy drinkers (>0 to <60 g/day in men and >0 to <40 g/day in women) have relatively low prevalence or risk of fatty liver compared to non- or seldom drinkers, with heterogeneity in the association based on sex and weight status [10], [13]. Therefore, the role of moderate alcohol intake in fatty liver pathogenesis remains to be confirmed.

The associations reported in previous studies have been inconsistent. One of the sources of the inconsistency is likely to be confounding due to other dietary factors. The association between alcohol intake and fatty liver may reflect healthy or unhealthy dietary habits, partly because alcoholic beverages are consumed in certain social or dietary settings in a population-specific manner [20]. A previous study in France showed that moderate drinkers had higher intake of vitamin C and dietary fiber than non- or heavy drinkers [21]. Another study showed that moderate alcohol consumers had high-quality diets, indicated by high consumption of fruit, vegetables, fish and low-fat meat [22]. A large number of dietary pattern analyses in Japan and elsewhere have shown that specific dietary patterns are associated with alcohol consumption [20], [23], [24]. Moreover, studies showed that dietary patterns characterized by high alcohol consumption were associated with the fatty liver prevalence [25], [26]. To date, however, no study has examined the association between alcohol intake and prevalence of fatty liver by accounting for underlying dietary patterns.

Here, we aimed to evaluate Japanese adults at risk of developing fatty liver diseases and assess the cross-sectional association of alcohol intake with fatty liver disease by treating dietary patterns as potential confounders.

## 2 Methods

### 2.1 Study design and participants

We conducted a cross-sectional study of medical check-up examinees. Details of the recruitment of participants for this study have been reported elsewhere [27]. Participants were recruited at the Center for Preventive Medicine at St. Luke's International Hospital in Tokyo, Japan. One month before their scheduled medical check-up (from January to March 2015), potential participants received documents by postal mail informing them of this study, including an introductory document on the study objectives and dietary questionnaires. Of the 9870 eligible participants (4758 men and 5112 women), 6823 (3163 men and 3660 women) consented to participate in this study. Informed consent was confirmed by participants' response to the questionnaires. This study was conducted under approval from the institutional review boards of Ochanomizu University and St. Luke's International Hospital.

Participants were excluded according to the following exclusion criteria: <30 years of age or >79 years of age (n = 163); possible pregnancy (n = 5); non-Japanese (n = 32); presence of other liver diseases (alcoholic fatty liver, hepatitis B, hepatitis C, liver cirrhosis, and liver cancer), gastrectomy or medication affecting fatty liver (n = 1677); incomplete or logical errors in responses to the dietary questionnaire (n = 53); possible over/under reporting of energy intake (less than half of or more than 1.5 times the estimated energy requirement for the lowest or highest physical activity level, respectively [28]) (n = 197); and consumption of a special diet prescribed by a health professional (n = 117). As a result, 4579 participants (2285 men and 2294 women) were included in the analysis.

### 2.2 Dietary assessment

Dietary intake was assessed using the brief-type self-administered diet history questionnaire (BDHQ). The validity of the BDHQ was assessed in previous studies [29], [30]. Alcohol intake was calculated based on drinking frequency and the amount of alcohol per drink for alcoholic beverages (beer; sake; shochu or shochu mixed with water or a carbonated beverage; whiskey; and wine) [29]. Pearson correlation coefficients between alcohol intake (% of energy; %E) estimated from the BDHQ and dietary records were 0.83 in men and 0.86 in women (unpublished observations, S. Kobayashi [30]). Frequency of consuming breakfast (<1 day/week; 1–2 days/week; 3–4 days/week; 5–6 days/week; everyday) was also assessed using one item in the BDHQ. The BDHQ covered the frequency of consumption of 55 food items. These food items were grouped into 32 food groups (Supplemental Table S1), and dietary patterns were evaluated for the use of statistical adjustment for dietary patterns in our primary analysis.

### 2.3 Ascertainment of fatty liver

Abdominal ultrasonography was performed at the routine health check-up [31]. The apparatuses used in the health check-up were Xario XG SSA-680A, Aplio400 TUS-A400, and Aplio500 TUS-A500 (Canon Medical Systems, Tochigi, Japan). The examinations were conducted by trained ultrasound technicians, with findings reviewed by board-certified radiologists and physicians for ultrasonography. These assessors were blinded to the participants' responses to the BDHQ. Fatty liver was ascertained based on the following four common criteria according to the guidelines in Asia pacific region [32], [33]: bright liver (high level echoes from the hepatic parenchyma), greater echogenicity in the liver than kidneys (sonographic contrast between the liver and right renal), deep attenuation (attenuation of the echo penetration into deep portion of the right hepatic lobe), and vascular blurring (blurring of the hepatic vein trunk). Accuracy of the criteria were verified in the previous studies [34], [35], [36], [37]. Participants were diagnosed with fatty liver by observing, at minimum, bright liver and hepatorenal contrast [35], [36].

### 2.4 Other variables

Height, body weight, HbA1c, hepatitis B antigen, and hepatitis C antibody (used for participant exclusion) were objectively measured at the health check-up. Body mass index (BMI) was calculated by dividing body weight (kg) by the square of the height (m^2^). Medical history, current treatments, smoking habit (current-smoker; ex-smoker; never smoker), and habitual exercise (almost never; 1–2 days/week; 3–5 days/week; everyday) were self-reported using a medical interview sheet. Habitual exercise was defined as the number of days per week spent doing 20 min or more of sports or walking.

### 2.5 Statistical analysis

Alcohol intake was expressed as a caloric density (%E) to account for potential confounding by total energy intake [38]. Participants were categorized into non-drinkers (0%E) and four drinkers' categories based on quartiles of alcohol intake. Associations of these intake categories with covariates were evaluated using a chi squared-test for categorical variables and regression analysis with assignment of median values to each category for continuous variables.

In this study, dietary patterns were treated as confounders for the association of alcohol consumption with fatty liver [20]. Dietary pattern variables were obtained using maximum likelihood factor analysis. First, 32 food group variables were log-transformed, log (x + 1), to improve the normality of the distributions and adjusted for energy intake using the residual method. Second, factor analysis was performed, followed by a scree plot to determine the optimal number of factors to retain, and varimax rotation was applied to facilitate interpretability of the factor scores.

The association of alcohol intake with fatty liver prevalence was examined by performing multivariable-adjusted Poisson regression modeling with robust standard error estimation [39], and estimating prevalence ratios (PRs) and 95% confidence intervals (95%CIs) for alcohol categories, with the non-drinkers category as the reference. To assess a dose-response relationship, quadratic models were also fitted. The non-linearity was tested by comparing the fitness (likelihood ratio test) of the models with and without the linear and squared terms of alcohol intake. In the preliminary analyses, we identified inconsistent results between categorical and continuous analyses for alcohol and identified implausibly high alcohol intake (up to 65%E from alcohol), which may have caused spurious findings in continuous analysis. To minimize the influence of such outliers, we conducted post hoc analyses after excluding outliers for alcohol intake, which were separately defined according to sex-specific statistics (the third quartile plus 1.5-times the interquartile range) in the analyses for linear and non-linear associations.

All models were analyzed with statistical adjustment for selected covariates. We accounted for biological plausibility of confounding and changes in point estimates (with 10% cutoff when removed from most adjusted models) to adjust for potential confounders and to achieve a parsimonious model [40], evaluating age (years), smoking status, habitual exercise, and weekly frequency of breakfast consumption. In the model that adjusted for these covariates, BMI was also included (kg/m^2^, linear and squared terms) because we speculated that adiposity would both confound and mediate the potentially causal association of alcohol consumption with fatty liver. A quadratic function was selected for BMI given the substantial impact of the adjustment on PRs for alcohol categories. In the multivariate model with or without BMI, we further adjusted for factor scores, representing overall dietary patterns as continuous variables, to assess confounding of dietary patterns in the association between alcohol intake and fatty liver.

As secondary analysis, we used alcohol intake expressed in grams/day rather than %E. To control for confounding due to overall diet and to avoid the effects of subjective decisions in the dietary pattern analysis, we performed an additional regression analysis by replacing factor scores with 27 food groups in a model that potentially included too many variables. Additionally, analyses stratified by sex and BMI categories (<23.0 and ≥ 23 kg/m^2^) were also conducted. Recognizing the limited evidence for the negative association of wine consumption with the likelihood of having NAFLD [41], we also conducted post-hoc analysis of the associations of different types of alcoholic beverages with fatty liver prevalence.

All analyses were performed using SPSS version 24 (IBM, Armonk, New York, United States). P values < 0.05 based on two-sided test statistics were considered statistically significant.

## 3 Results

Non-drinkers accounted for 19.7% of participants (902/4579). Shochu contributed to alcohol intake the most (29.4%) followed by beer (25.9%), wine (18.9%), sake (14.0%), and whisky (11.8%). There were significant differences in gender, age category, BMI category, smoking status, and habitual exercise among the categories of alcohol intake (Table 1). In general, food intake was negatively associated with categories of alcohol intake (Table 2). However, intake of noodles, coffee, and shellfish increased according to categories of alcohol intake.

**Table 1:** Population characteristics according to alcohol consumption in 4579 adults enrolled in St Luke's International Hospital's annual health check-up program in Japan.

	Non-drinkers	Quartiles among drinkers				P^a^
	n = 902	Q1 (n = 930)	Q2 (n = 917)	Q3 (n = 914)	Q4 (n = 916)	
Alcohol intake, %E, median (range)	0.0	0.5 (0.0–1.5)	3.3 (1.6–5.5)	8.5 (5.6–12.4)	18.9 (12.5–64.7)	
Sex, % men	27.4	35.2	51.6	63.5	71.8	<0.001
Age (years), mean (SD)	54.2 (11.8)	51.9 (11.1)	51.4 (10.8)	52.2 (10.5)	51.1 (9.7)	<0.001
BMI (kg/m^2^), mean (SD)	22 (3.5)	21.7 (3.1)	22.1 (3.0)	22.7 (3.2)	23.3 (3.1)	<0.001
Smoking status, %						
Never	75.5	77.4	64.3	54.0	38.2	<0.001
Ex-smoker	18.3	17.6	26.5	34.4	38.4	
Current smoker	6.2	4.9	9.2	11.6	23.4	
Habitual exercise, %						
Everyday	10.6	9.1	10.3	11.2	7.9	0.003
3–5 days/week	22.0	21.2	20.8	20.5	17.7	
1–2 days/week	33.8	37.4	39.4	41.2	42.7	
Almost never	33.6	32.3	29.6	27.1	31.8	
Frequency of eating breakfast, %						
Everyday	61.5	61.6	56.6	50.8	40.4	<0.001^b^
5–6 days/week	10.1	10.6	10.5	14	10.3	
3–4 days/week	8.3	7.8	8.8	10.5	11.9	
1–2 days/week	8.9	8.6	12.1	10.4	13.5	
<1 day/week	10.5	11.1	11.3	13.8	23.8	
Missing	0.7	0.2	0.7	0.5	0.1	

BMI, body mass index; SD, standard deviation; %E, % of energy.

a Based on regression analysis with assignment of median values to each category for continuous variables and chi squared-test for categorical variables.

b Individuals with missing values were included in the “Everyday” category.

**Table 2:** Consumption of major individual foods and dietary pattern scores according to alcohol consumption in 4579 adults enrolled in St Luke's International Hospital's annual health check-up program in Japan.^a^

	Non-drinkers	Quartiles among drinkers				P for trend^b^
	n = 902	Q1 (n = 930)	Q2 (n = 917)	Q3 (n = 914)	Q4 (n = 916)	
Alcohol intake, %E median (range)	0.0	0.5 (0.02–1.5)	3.3 (1.6–5.5)	8.5 (5.6–12.4)	18.9 (12.5–64.7)	
Nutrient intake						
Dietary fiber, g/day	12.8 (4.3)	12.2 (3.8)	11.7 (3.5)	10.7 (3.5)	8.7 (3.1)	<0.001
Carbohydrates, %E	54.2 (6.4)	53.6 (6.3)	51.6 (6.3)	48.7 (6.5)	41.6 (7.5)	<0.001
Total fat, %E	28.5 (4.9)	28.6 (4.7)	28.2 (4.9)	26.3 (4.9)	23.1 (5.2)	<0.001
Saturated fat, %E	7.7 (1.7)	7.7 (1.6)	7.5 (1.6)	6.9 (1.5)	5.8 (1.5)	<0.001
Sodium, mg/day	4206 (735)	4185 (712)	4148 (700)	4123 (794)	3854 (766)	<0.001
Food consumption, g/day						
Rice	222 (104)	230 (106)	223 (108)	215 (112)	107 (98)	<0.001
Noodles	62.6 (46.2)	62.1 (41.8)	67.4 (46.0)	71.8 (49.9)	72.1 (52.9)	<0.001
Bread	41.0 (27.7)	41.5 (24.7)	37.6 (25.9)	32.7 (25.7)	22.4 (23.3)	<0.001
Pulses	73.9 (45.2)	72.2 (44.2)	67.0 (39.2)	62.4 (41.7)	55.2 (38.9)	<0.001
Potatoes	44.8 (38.0)	40.3 (35.9)	37.5 (32.3)	32.8 (33.1)	22.5 (26.8)	<0.001
Sugar	4.3 (3.7)	4.2 (3.4)	3.8 (2.9)	3.3 (2.7)	3.0 (2.8)	<0.001
Confectionary	81.5 (45.8)	80.7 (42.7)	74.0 (39.0)	61.0 (37.9)	45.8 (31.6)	<0.001
Oil	15.9 (6.6)	16.7 (6.8)	17.2 (7.2)	16.9 (6.9)	15.5 (7.1)	0.002
Fruit	117.3 (83.2)	104.7 (71.9)	99.1 (72.4)	81.9 (67.4)	60.9 (60.8)	<0.001
Green/yellow vegetables	112.1 (70.9)	106.1 (62.0)	102.7 (62.4)	93.1 (60.9)	76.0 (54.3)	<0.001
Other vegetables	156.6 (84.6)	148.2 (76.1)	142.7 (75.5)	136.7 (75.1)	113.9 (64.8)	<0.001
Pickled vegetables	13.9 (16.1)	12.7 (14.5)	11.2 (12.9)	12.1 (13.3)	11.5 (12.5)	0.006
Mushrooms	13.3 (10.1)	12.9 (9.4)	12.1 (9.2)	11.2 (8.8)	9.4 (8.4)	<0.001
Seaweed	11.5 (11.0)	11.3 (10.0)	10.4 (9.4)	10.2 (9.4)	8.7 (9.4)	<0.001
Green tea	264 (221)	246 (205)	222 (196)	210 (196)	196 (198)	<0.001
Black/oolong tea	116 (149)	111 (144)	112 (146)	98.8 (142)	86.0 (136)	<0.001
Coffee	223 (179)	245 (179)	257 (180)	270 (180)	251 (185)	0.011
Soft drink	32.1 (78.9)	32.7 (82.0)	29.0 (74.0)	24.9 (71.9)	26.5 (73.9)	0.038
Fruit/vegetable juice	52.0 (92.4)	55.9 (87.6)	55.4 (92.6)	53.6 (92.7)	51.3 (91.7)	0.42
Sea products	32.1 (23.2)	31.6 (24.6)	29.2 (21.0)	29.3 (22.8)	25.9 (18.5)	<0.001
Other fish	33.8 (24.9)	32.3 (23.0)	31.9 (23.4)	30.9 (23.5)	29.1 [23]	<0.001
Shellfish	11.9 (12.5)	12.7 (13.7)	13.7 (14.7)	14.7 (16.1)	14.3 (17.5)	<0.001
Chicken	33.5 (21.2)	34.3 (19.1)	36.6 (20.6)	34.9 (20.1)	35.1 (20.0)	0.35
Pork and beef	36.9 (22.5)	38.2 (20.8)	37.3 (21.3)	36.2 (22.0)	32.4 (18.8)	<0.001
Processed meats	9.3 (8.1)	9.7 (8.2)	10.0 (8.0)	9.8 (8.4)	8.4 (7.3)	<0.001
Egg	34.9 (21.5)	33.2 (20.9)	34.6 (21.5)	33.7 (22.5)	30.6 (23.6)	<0.001
Dairy products	132.6 (91.9)	122.9 (86.8)	119.3 (89.0)	111.1 (90.4)	81.6 (84.1)	<0.001
Dietary pattern (factor scores) c						
“Plant foods”	0.15 (1.09)	0.04 (0.98)	0.02 (0.97)	−0.03 (0.97)	−0.18 (0.96)	<0.001
“Bread and confectionary”	0.90 (0.58)	0.60 (0.54)	0.13 (0.60)	−0.38 (0.65)	−1.24 (0.86)	<0.001
“Fish”	0.12 (1.09)	0.10 (0.94)	−0.01 (0.97)	−0.02 (0.97)	−0.18 (1.00)	<0.001
“Oils and meats”	−0.18 (1.07)	0.03 (0.95)	0.16 (0.94)	0.09 (0.95)	−0.11 (1.05)	0.18

%E, % of energy.

a All values are mean (standard deviation) unless otherwise indicated.

b Based on regression analysis with assignment of median values of alcohol intake to each category.

c By design of factor analysis, means and standard deviations were scaled to 0.0 and 1.0, respectively, in the overall study population. Four factor scores were arbitrarily established according to high factor loading values assigned to specific food groups (Figure S1).

Four factors (dietary patterns) were derived from factor analysis (Supplemental Fig. 1): the first was characterized mainly by high intake of fruit, vegetables, mushrooms, seaweed and soy-based foods (“Plant foods”); the second was characterized by high intake of bread, confectionary and fruit and low intake of alcoholic beverages (“Bread and confectionary”); the third was characterized by high intake of fish, pickled vegetables, mushrooms and seaweed (“Fish”); and the fourth was characterized by high intake of oils and meats (“Oils and meats”). Except for “Oils and meats”, higher dietary pattern scores were negatively associated with alcohol intake.

Fatty liver was ascertained in 24.5% (1120/4579) of participants. Analyses including potential outliers of alcohol intake (%E of alcohol up to 64%) indicated that moderate drinkers (%E of alcohol 0.1%–12%) had 20%–30% lower prevalence of fatty liver than non-drinkers after adjustment for potential confounders and dietary patterns (Table S2). Adjustment for BMI and continuous assessment for linear and non-linear associations demonstrated no significant associations.

**Fig. 1:**
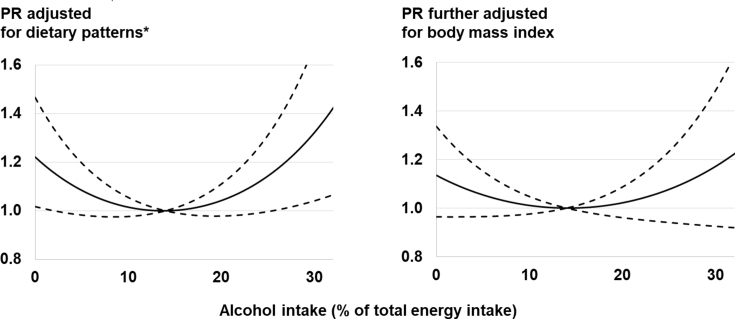
Association of alcohol intake with prevalence of fatty liver among 4319 adults enrolled in St Luke's International Hospital's annual health check-up program in Japan. Solid and dotted lines indicate prevalence ratios (PR) and 95% confidence intervals, respectively. The reference (PR = 1.0) was 13% energy from alcohol (horizontal axis), an integer indicating the lowest prevalence in the population. *P = 0.02 and 0.003 for the overall association of alcohol and a non-linear trend, respectively, when adjusted for age, sex, physical activity, smoking status, and four dietary pattern variables (left); and P = 0.17 and 0.06, respectively, after further adjustment for body mass index (right).

Based on sex-specific definitions of alcohol intake (≥32.26%E for men and ≥14.5%E for women), 54 men and 206 women were identified as outliers or over-reporters. The results after excluding these participants are presented in Table 3. Adjustment for potential confounders other than BMI and dietary patterns revealed a non-linear significant association of alcohol intake with prevalence of fatty liver (p for non-linear trend = 0.02). Moreover, moderate drinkers (≥1.36 to ≤4.74 %E) had a significantly lower prevalence of fatty liver than non-drinkers, with a PR (95% CI) of 0.81 (0.68–0.97). Further adjustment for dietary patterns revealed that the prevalence of fatty liver was lower among low to moderate drinkers than non-drinkers, with PRs of 0.87 (0.72–1.03), 0.73 (0.61–0.88), 0.72 (0.59–0.86), and 0.77 (0.62–0.95) for intake quartiles 1–4 among drinkers (p for non-linear trend = 0.003). Models that further adjusted for linear and squared terms for BMI showed that the association was attenuated toward the null. Although a significant U-shaped association was observed after adjustment for lifestyle factors (p for non-linearity = 0.02) and dietary patterns (p = 0.003), confidence intervals became wide and the association was not significant after further adjustment for BMI (p = 0.09 and 0.06, respectively) (Fig. 1).

**Table 3:** Association of alcohol consumption with prevalence of fatty liver in 4319 adults enrolled in St Luke's International Hospital's annual health check-up program in Japan.

	Non-drinkers	Quartiles among drinkers				P for non-linear trend^a^	P for overall association^a^
		Q1	Q2	Q3	Q4		
Alcohol intake (%E), median (range)	0.0	0.46 (0.02–1.35)	2.81 (1.36–4.74)	7.21 (4.75–10.5)	15.2 (10.6–32.2)		
N cases/N participants	188/902	172/860	176/841	216/855	324/861		
Prevalence ratio (95% CI)							
Adjusted for selected covariates b	1.00 (reference)	0.91 (0.77, 1.09)	0.81 (0.68, 0.97)	0.85 (0.72, 1.00)	1.01 (0.86, 1.18)	0.02	0.02
+ dietary patterns^c^	1.00 (reference)	0.87 (0.72, 1.03)	0.73 (0.61, 0.88)	0.72 (0.59, 0.86)	0.77 (0.62, 0.95)	0.003	0.02
+ BMI d	1.00 (reference)	1.05 (0.91, 1.23)	0.96 (0.83, 1.12)	0.92 (0.79, 1.06)	1.00 (0.88, 1.15)	0.09	0.24
+ dietary patterns and BMI^c^,^d^	1.00 (reference)	1.04 (0.89, 1.21)	0.94 (0.80, 1.10)	0.87 (0.74, 1.03)	0.92 (0.76, 1.12)	0.06	0.17

BMI, body mass index; 95% CI, 95% confidence interval; %E, % of energy.

a P values were based on likelihood ratio tests for both linear and quadratic terms of alcohol consumption, derived from the post hoc analysis, which excluded 260 participants who reported alcohol intake greater than the sex-specific definition of third quartile plus 1.5 × interquartile range (≥32.26%E for men and ≥14.5%E for women). P for linear trends was 0.19, 0.006, 0.14, and 0.06 for the four models. Without the exclusion of outliers, no associations were significant (Table S2 and S3) (p > 0.1).

b Adjusted for age (years, continuous), sex, smoking habit (current-smoker; ex-smoker; never smoker), and habitual exercise (almost never; 1–2 days/week; 3–5 days/week; everyday).

c Additionally adjusted for the four factor scores for the four dietary patterns.

d Additionally adjusted for linear and squared terms for BMI.

The results were materially similar when we modelled 27 food groups simultaneously as covariates and when we modelled alcohol consumption in grams/day instead of %E (P for non-linearity = 0.10 and 0.034, respectively, after adjustment for BMI; Table S3 and S4). Stratified analyses for sex and BMI, where the number of cases was <844 in each stratum, showed that the association of alcohol intake with prevalence of fatty liver was not significant in any strata (Table S5), but suggested that prevalence was more pronounced in women and in non-overweight adults (BMI<23 kg/m^2^) than their respective counterparts. In the analysis by the alcoholic beverages, while point estimates varied and indicated U-shaped associations for wine only, none of the associations was significant (P > 0.05 for each set of linear and quadratic terms for different beverages; Table S6).

## 4 Discussion

To our knowledge, this is the first study to demonstrate a U-shaped association between alcohol intake and fatty liver prevalence after adjustment for underlying dietary patterns. Additionally, the observed association was sensitive to the reporting of excessive alcohol intake and was partly explained by obesity. Our findings suggest that studies on the prevention of fatty liver should examine obesity status and a range of alcohol intake categories rather than simply dichotomizing alcohol intake.

The association between alcohol intake and fatty liver was no longer significant after adjustment for BMI, with the quadratic curve becoming imprecise (wide 95% confidence interval). While this study could not discern BMI as a confounder or mediator, the findings provide a clinical implication that both obesity and alcohol consumption should be targeted for adults at high risk of fatty liver or other diseases. While studies have examined the longitudinal association between alcohol intake and fatty liver onset [8], [16], [17], the effect of BMI and other adiposity measures on this association has not been clarified. In both population and clinical settings, further prospective studies are warranted to evaluate the relationship between alcohol intake and fatty liver diseases by considering changes in BMI, waist circumference, and other anthropometric measures.

Alcohol intake has been recognized as a key component in an overall dietary quality in adults. In our study population, higher alcohol intake (assessed using %E) was negatively associated with several dietary variables including both healthy and unhealthy items such as fish, fruit, vegetables, and confectionary. Previous studies have suggested that high intake of eicosapentaenoic acid and docosahexaenoic acid [42], magnesium [43], and vitamin C [44] is linked to low prevalence of fatty liver diseases. In contrast, consumption of refined carbohydrates may increase hepatic de novo lipogenesis and risk of fatty liver diseases [45]. Based on this existing knowledge, our finding that there were no notable differences before and after adjustment for dietary pattern scores indicates that dietary patterns may be confounding in both positive and negative ways, rather than dietary patterns not being a confounder. An intervention or behavioral changes to reduce excess alcohol intake may therefore require understanding a person's accompanying dietary habits.

Excluding outliers of alcohol intake also considerably changed our results despite calibration of the estimated alcohol intake to account for observed over-reporters [29]. In contrast, total energy intake is well-known to be under-reported [46]. Therefore, the presence of both over-reporting of alcohol intake and under-reporting of energy intake may have resulted in an excess of alcohol consumers in our study. Despite this, a non-linear association was detected in our secondary analysis, which examined alcohol intake expressed in grams/day. Detailed analysis of the unstable findings due to the presence of an excess of alcohol consumers is therefore required, particularly on the dietary patterns linked to total energy intake and alcohol intake. Because of potential errors in our dietary assessment, we did not determine cut-off points for identifying moderate or excess intake of alcohol. This and our finding of a possible non-linear association lend support to our conclusion that consumers of excess alcohol require special attention in both clinical and research settings.

The strengths of this study include dietary pattern analysis and characterization of dose-response relationships between alcohol intake and fatty liver prevalence with adjustment for dietary patterns and energy intake, which have not been accounted for in previous studies.

Several limitations of this study warrant discussion. First, due to the cross-sectional design, causality of the relationships between alcohol intake and fatty liver could not be ascertained. Therefore, analysis that includes non-drinkers might lead to “sick-quitter bias” whereby participants may have decreased their alcohol intake for health-related reasons [47] despite exclusion of those with prevalent diseases or medications. However, while our findings related to non-drinkers should be interpreted with caution, the (U-shaped) association among consumers may be free from this bias. Future longitudinal research is warranted to replicate our findings after accounting for dietary patterns. Second, there may have been residual confounding due to unmeasured or imprecisely measured covariates. The lack of socioeconomic variables, such as educational level, occupation, and family income, may be crucial because of the greater likelihood that individuals with a high socioeconomic background reported moderate alcohol intake and were less likely to have fatty liver than low alcohol consumers. However, comparison between moderate- and high-consumers may be less concerning in this regard. Third, fatty liver assessment based on abdominal ultrasonography was suboptimal compared to liver biopsy. Although ultrasonography allows for reliable and accurate identification of moderate-severe fatty liver (≥20–30% fat infiltration) [37], quantification of fat infiltration to the liver with magnetic resonance imaging (MRI) outperforms ultrasonography to detect ≥5% fatty liver [48]. Nevertheless, use of the ultrasonography is justifiable and unlikely to produce substantial bias in this work. There is the global consensus for using ultrasonography as the first-line examination to detect fatty liver in daily clinical practice because of its low cost, low invasiveness, and wide availability [49]. Use of MRI would be practically not feasible because of its resource-intensiveness for our clinical setting whether thousands of adults undergo clinical examinations every year. Moreover, bias due to misclassification of fatty liver diagnosis is unlikely to have existed because dietary habits were uninformed to assessors of ultrasonography results and because of no credible source of differential misclassification. Our results might be imprecise, elevating standard errors, but still accurate for the shape of the association. Fourth, although the dietary history questionnaire used in this study has been assessed for validity for ranking of alcohol intake [30], the estimated amount of habitual alcohol intake is likely to have been inaccurate. As mentioned above, this study was not designed to determine the absolute levels of alcohol that would be important to consider a clinical cutoff. Similarly, the questionnaire may have limited ability to assess health effects of different types of alcoholic beverages because, while total alcohol intake might be assessed reasonably well [30], consumption of each alcoholic beverage was assessed with a single question with discrete categories only, resulting in too crude quantitative data. Fifth, our sample size was limited, thereby limiting the power to detect interactions or effects of different types of alcoholic beverages that mutually interrelated in this study. Therefore, the potential effect of alcohol intake on fatty liver could not be accurately assessed according to sex or weight status [10] or by alcoholic beverage [41]. Finally, the generalizability of our results to other populations may be limited. Participants in this study likely represent a general middle-aged occupational population in Japan because around 80% of our participants were referred to the health check-up center via employer-sponsored programs. In Japan and elsewhere, populations at high risk of obesity or metabolic diseases and those with low socioeconomic status (e.g. without a full-time job) should be examined for their alcohol intake and related dietary patterns.

In conclusion, we found a non-linear, U-shaped association of alcohol intake with prevalence of fatty liver. While alcohol intake was associated with consumption of many food items and dietary patterns, the association is likely to have been present regardless of underlying dietary habits. The association was not stable when we included participants who reported intake of excessive levels of alcohol relative to their total energy intake. Moreover, the association was partly explained by the obesity status.

## Conflict of interest statement and funding sources

The authors have no conflicts of interest to declare. This work was supported by the 10.13039/501100001691Japan Society for the Promotion of Science (JSPS) KAKENHI (R.T. grant number 14J11939 and K.I. grant number 23240104). JSPS had no role in the design, analysis or writing of this article. F.I. was supported by an 10.13039/501100000265MRC Epidemiology Unit Core Grant (MC_UU_12015/5).

## Authors' contributions

R. Tajima carried out the questionnaire survey, analyzed the data, and wrote the initial draft of the manuscript. F. Imamura contributed to analysis and interpretation of data, and assisted in the preparation of the manuscript. T. Kimura, K. Masuda, and K. Iida played leading roles in the conception and design of the study. S. Kobayashi gave various opinions on interpretation of the study results regarding the validity of the dietary questionnaire. All study members reviewed the article and provided final approval of the version to be published. All of the authors certify that they have participated sufficiently in the work.
