# Supplementary material for: Association of alcohol consumption with prevalence of fatty liver after adjustment for dietary patterns: Cross-sectional analysis of Japanese middle-aged adults
Source: Clin Nutr. 2020 May;39(5):1580–6. doi: 10.1016/j.clnu.2019.07.001 (PMC7218709; doi:10.1016/j.clnu.2019.07.001)
Supplement: Multimedia component 1 [file mmc1.docx]

**Table S1. Food grouping for factor analysis.**

|  | Food group in factor analysis | Food items in the dietary questionnaire (the brief-type self-administered diet history questionnaire) |
| --- | --- | --- |
| Grain products | Rice | Rice |
|  | Noodles | Buckwheat noodles; Japanese wheat noodles; Instant noodles and Chinese noodles; Spaghetti and macaroni |
|  | Bread | Bread |
|  | Pulses | Tofu and tofu products; Natto; Miso for miso soup |
|  | Potatoes | Potatoes |
| Fruit, vegetables | Fruit | Citrus fruit including oranges; Strawberries, persimmons and kiwi fruit; Other fruit |
|  | Green and yellow vegetables | Carrots and pumpkins; Tomatoes, tomato ketchup, boiled tomato and stewed tomato; Green leafy vegetables including broccoli |
|  | Other vegetables | Raw vegetables used in salad (cabbage and lettuce); Cabbage and Chinese cabbage; Radishes and turnips; Other root vegetables (onions, burdock and lotus root) |
|  | Mushrooms | Mushrooms |
|  | Seaweed | Seaweed |
|  | Pickled vegetables | Salted green and yellow vegetable pickles; Other salted vegetable pickles |
| Meat, fish | Chicken | Chicken (including ground chicken); Liver |
|  | Pork and beef | Pork and beef (including ground pork and beef) |
|  | Processed meats | Ham, sausages and bacon |
|  | Sea products | Dried fish and salted fish (including salted mackerel, salted salmon and dried horse mackerel); Small fish with bones; Canned tuna |
|  | Other fish | Oily fish (including sardines, mackerel, saury, amberjack, herring, eel and fatty tuna); Non-oily fish (including salmon, trout, white fish, freshwater fish and bonito) |
|  | Shellfish | Squid, octopus, shrimp and clams |
| Dairy, eggs | Dairy products | Full-fat milk and yoghurt; Low-fat milk and yoghurt |
|  | Eggs | Eggs |
| Sweets, condiments | Oils | Mayonnaise and salad dressing; Oil used for cooking |
|  | Sugar | Sugar for coffee and black tea; Sugar used for cooking |
|  | Confectionary | Rice crackers, rice cakes and Japanese-style pancakes; Japanese sweets; Cakes; Cookies and biscuits; Ice cream |
| Beverages | Green tea | Green tea |
|  | Black and oolong tea | Black and oolong tea (including other Chinese tea) |
|  | Coffee | Coffee |
|  | Soft drink | Cola and sweetened soft drinks (including sports drinks) |
|  | Fruit and vegetable juice | Fruit juice and vegetable juice |
|  | Beer | Beer |
|  | Sake | Sake |
|  | Shochu | Shochu and shochu mixed with water or a carbonated beverage |
|  | Whiskey | Whiskey |
|  | Wine | Wine |

| **Dietary variables** |  | **Factor 1** | **Factor 2** | **Factor 3** | **Factor 4** |  |  |  |
| --- | --- | --- | --- | --- | --- | --- | --- | --- |
| Grains, potatoes | Rice | -0.13 | 0.03 | -0.05 | -0.31 |  |  |  |
|  | Bread | -0.09 | 0.43 | -0.17 | 0.07 |  |  |  |
|  | Noodles | -0.32 | -0.07 | 0.05 | 0.05 |  |  |  |
|  | Pulses | 0.42 | 0.05 | 0.30 | -0.07 |  |  |  |
|  | Potatoes | 0.25 | 0.21 | 0.22 | 0.13 |  |  |  |
| Fruit, vegetables | Fruit | 0.32 | 0.36 | 0.20 | -0.07 |  |  |  |
|  | Green or yellow vegetables | 0.70 | 0.17 | 0.22 | 0.24 |  |  |  |
|  | Other vegetables | 0.73 | 0.10 | 0.26 | 0.28 |  |  |  |
|  | Mushrooms | 0.49 | 0.09 | 0.30 | 0.20 |  |  |  |
|  | Seaweed | 0.37 | 0.03 | 0.38 | 0.08 |  |  |  |
|  | Pickled vegetables | -0.01 | -0.02 | 0.30 | 0.00 |  |  |  |
| Meat, fish | Chicken | 0.01 | -0.13 | 0.00 | 0.35 |  |  |  |
|  | Pork and beef | -0.01 | -0.01 | -0.03 | 0.40 |  |  |  |
|  | Processed meats | -0.09 | 0.07 | 0.06 | 0.36 |  |  |  |
|  | Sea products | 0.08 | 0.01 | 0.52 | 0.02 |  |  |  |
|  | Other fish | 0.16 | -0.03 | 0.47 | 0.10 |  |  |  |
|  | Shellfish | -0.10 | -0.13 | 0.37 | 0.23 |  |  |  |
| Dairy, eggs | Dairy products | 0.17 | 0.29 | 0.04 | -0.05 |  |  |  |
|  | Eggs | 0.09 | 0.06 | 0.06 | 0.19 |  |  |  |
| Sweets, condiments | Oils | -0.06 | 0.02 | -0.09 | 0.63 |  |  |  |
|  | Sugar | 0.12 | 0.15 | 0.25 | -0.01 |  |  |  |
|  | Confectionary | -0.08 | 0.44 | -0.10 | 0.01 |  |  | **+0.5** |
| Beverages | Green tea | 0.06 | 0.08 | 0.16 | -0.01 |  |  |  |
|  | Black and oolong tea | 0.01 | 0.17 | 0.05 | 0.22 |  |  |  |
|  | Coffee | -0.03 | -0.02 | -0.10 | 0.07 |  |  |  |
|  | Soft drink | -0.34 | 0.06 | -0.02 | 0.10 |  |  |  |
|  | Fruit or vegetable juice | -0.07 | 0.07 | 0.04 | 0.04 |  |  | **0** |
|  | Sake | -0.05 | -0.31 | 0.04 | -0.11 |  |  |  |
|  | Beer | -0.18 | -0.50 | -0.10 | 0.03 |  |  |  |
|  | Shochu | -0.11 | -0.43 | -0.09 | -0.02 |  |  |  |
|  | Whiskey | -0.06 | -0.34 | -0.07 | 0.00 |  |  |  |
|  | Wine | 0.05 | -0.24 | -0.05 | 0.06 |  |  | **-0.5** |

**Figure S1**. Loading values of four factors derived from factor analysis of 32 food groups among 4,579 participants enrolled in St Luke’s International Hospital’s screening program (from January to March 2015) in Japan. Food grouping is shown in Table S1.

**Table S2. Association of alcohol intake with fatty liver in 4,579 adults enrolled in St Luke’s International Hospital’s annual health check-up program in Japan.**

|  | Non-drinkers | Quartiles among drinkers | | | | P for non-linear trend | P for overall association ^a^ |
| --- | --- | --- | --- | --- | --- | --- | --- |
|  |  | Q1 | Q2 | Q3 | Q4 |  |  |
| Alcohol intake, %E, median (range) | 0.0 | 0.5 (0.02–1.5) | 3.3 (1.6–5.5) | 8.5 (5.6–12.4) | 18.9 (12.5–64.7) |  |  |
| N cases/N total | 188/902 | 188/930 | 190/917 | 250/914 | 304/916 |  |  |
| Prevalence ratios (95% CI) |  |  |  |  |  |  |  |
| Adjusted for potential confounders ^b^ | 1.00 (reference) | 0.93 (0.78, 1.10) | 0.78 (0.66, 0.93) | 0.90 (0.77, 1.06) | 0.98 (0.84, 1.15) | 0.93 | 0.27 |
| + dietary patterns ^c^ | 1.00 (reference) | 0.88 (0.74, 1.05) | 0.70 (0.59, 0.84) | 0.76 (0.63, 0.91) | 0.75 (0.61, 0.94) | 0.25 | 0.25 |
| + BMI ^d^ | 1.00 (reference) | 1.05 (0.90, 1.22) | 0.93 (0.80, 1.09) | 0.97 (0.84, 1.11) | 0.96 (0.84, 1.10) | 0.74 | 0.67 |
| + dietary patterns + BMI ^cd^ | 1.00 (reference) | 1.03 (0.89, 1.20) | 0.90 (0.77, 1.06) | 0.91 (0.77, 1.07) | 0.87 (0.72, 1.06) | 0.42 | 0.33 |

^a^ Significance of the non-linear association was assessed based on the likelihood ratio test by comparing the model with and without the linear and squared terms for alcohol intake.

^b^ Adjusted for age (years), sex, smoking habit (current-smoker; ex-smoker; never smoker), and habitual exercise (almost never; 1–2 days/week; 3–5 days/week; everyday).

^c^ Additionally adjusted for the four factor scores for the four dietary patterns.

^d^ Additionally adjusted for linear and squared terms for BMI.

BMI, body mass index; 95% CI, 95% confidence interval; %E, % of energy.

**Table S3. Quadratic models with linear and squared terms for alcohol intake in adults enrolled in St Luke’s International Hospital’s annual health check-up program in Japan.**

|  | Including outliers (n cases=1,120, n total=4,579) | | | | | Excluding outliers (n cases=1,076, n total=4,319) | | | | |
| --- | --- | --- | --- | --- | --- | --- | --- | --- | --- | --- |
|  | Linear term  (per 10%E) | p | Squared term  (per 10%E^2^) | p | P for likelihood ratio test | Linear term  (per 10%E) | p | Squared term  (per 10%E^2^) | p | P for likelihood ratio test |
| Alcohol intake, %E (range) | (0.0–64.7) |  | - |  |  | (0.0–32.2) |  | - |  |  |
| Prevalence ratios (95% CI) |  |  |  |  |  |  |  |  |  |  |
| Adjusted for potential confounders ^b^ | 1.03 (0.91, 1.17) | 0.60 | 1.00 (0.97, 1.04) | 0.93 | 0.27 | 0.88 (0.73, 1.06) | 0.19 | 1.08 (1.01, 1.16) | 0.02 | 0.02 |
| + dietary patterns ^c^ | 0.89 (0.76, 1.04) | 0.16 | 1.02 (0.98, 1.06) | 0.25 | 0.25 | 0.75 (0.61, 0.92) | 0.006 | 1.11 (1.04, 1.19) | 0.003 | 0.02 |
| + BMI ^d^ | 0.97 (0.87, 1.08) | 0.61 | 1.01 (0.97, 1.04) | 0.74 | 0.67 | 0.88 (0.75, 1.04) | 0.14 | 1.06 (0.99, 1.13) | 0.09 | 0.24 |
| + dietary patterns and BMI ^cd^ | 0.91 (0.79, 1.05) | 0.21 | 1.01 (0.98, 1.05) | 0.42 | 0.33 | 0.84 (0.69, 1.01) | 0.06 | 1.07 (1.00, 1.14) | 0.06 | 0.17 |
| +27 food groups | - | - | - | - | - | 1.04 (0.81, 1.33) | 0.75 | 1.09 (1.02, 1.17) | 0.016 | 0.006 |
| +27 food groups and BMI ^d^ | - | - | - | - | - | 0.92 (0.74, 1.14) | 0.46 | 1.06 (0.99, 1.13) | 0.10 | 0.23 |

^a^ Outliers for alcohol intake were separately defined according to sex as follows: more than the third quartile plus 1.5-times the interquartile range (≥32.26%E for men and ≥14.5%E for women).

^b^ Adjusted for age (years), sex, smoking habit (current-smoker; ex-smoker; never smoker), and habitual exercise (almost never; 1–2 days/week; 3–5 days/week; everyday).

^c^ Additionally adjusted for the four factor scores for the four dietary patterns.

^d^ Additionally adjusted for linear and squared terms for BMI.

BMI, body mass index; 95% CI, 95% confidence interval; %E, % of energy.

**Table S4. Quadratic models with linear and squared terms for alcohol intake in adults enrolled in St Luke’s International Hospital’s annual health check-up program in Japan (n cases=991, n total=4,079). ^a^**

|  | Linear term  (per 10 g/day) | p | Squared term  (per 10 g/day^2^) | p | P for likelihood ratio test |
| --- | --- | --- | --- | --- | --- |
| Alcohol intake, g/day (range) | (0.0–62.5) |  | - |  |  |
| Prevalence ratios (95% CI) |  |  |  |  |  |
| Adjusted for potential confounders ^b^ | 0.91 (0.82, 1.00) | 0.055 | 1.02 (1.01, 1.04) | 0.011 | 0.028 |
| + dietary patterns ^c^ | 0.82 (0.73, 0.92) | 0.001 | 1.03 (1.01, 1.05) | 0.001 | 0.006 |
| + BMI ^d^ | 0.92 (0.85, 1.00) | 0.065 | 1.02 (1.00, 1.03) | 0.044 | 0.15 |
| + dietary patterns and BMI ^cd^ | 0.90 (0.81, 0.99) | 0.038 | 1.02 (1.00, 1.04) | 0.034 | 0.12 |

^a^ Outliers for alcohol intake were separately defined according to sex as follows: more than the third quartile plus 1.5-times the interquartile range (≥62.5 g/day for men and ≥23.8 g/day for women). Five hundred participants were excluded from the analysis.

^b^ Adjusted for age (years), sex, smoking habit (current-smoker; ex-smoker; never smoker), habitual exercise (almost never; 1–2 days/week; 3–5 days/week; everyday) and energy intake (kcal/day).

^c^ Additionally adjusted for the four factor scores for the four dietary patterns.

^d^ Additionally adjusted for linear and squared terms for BMI.

BMI, body mass index; 95% CI, 95% confidence interval.

**Table S5. Stratified analyses for the association of alcohol intake with fatty liver in 4,319 adults enrolled in St Luke’s International Hospital’s annual health check-up program in Japan. ^a^**

|  | N cases/N total | Linear term  (per 10% energy) | p | Squared term  (per 10% energy^2^) | p | P for likelihood ratio test |
| --- | --- | --- | --- | --- | --- | --- |
| Stratified analysis for sex |  |  |  |  |  |  |
| Men | 833/2231 |  |  |  |  |  |
| Alcohol intake, %E (range) |  | (0.0–32.2) |  | - |  |  |
| Prevalence ratios (95% CI) |  |  |  |  |  |  |
| Adjusted for potential confounders ^b^ |  | 0.92 (0.76, 1.12) | 0.41 | 1.06 (0.99, 1.15) | 0.09 | 0.04 |
| + dietary patterns ^c^ |  | 0.81 (0.65, 1.01) | 0.06 | 1.08 (1.01, 1.16) | 0.03 | 0.12 |
| + BMI ^d^ |  | 0.91 (0.77, 1.08) | 0.30 | 1.05 (0.98, 1.12) | 0.18 | 0.32 |
| + dietary patterns and BMI ^cd^ |  | 0.86 (0.71, 1.05) | 0.15 | 1.05 (0.98, 1.13) | 0.13 | 0.30 |
| Women | 243/2088 |  |  |  |  |  |
| Alcohol intake, %E (range) |  | (0.0–14.5) |  | - |  |  |
| Prevalence ratios (95% CI) |  |  |  |  |  |  |
| Adjusted for potential confounders ^b^ |  | 0.65 (0.24, 1.78) | 0.40 | 1.49 (0.62, 3.59) | 0.37 | 0.43 |
| + dietary patterns ^c^ |  | 0.36 (0.12, 1.10) | 0.073 | 1.91 (0.79, 4.61) | 0.15 | 0.09 |
| + BMI ^d^ |  | 0.81 (0.34, 1.89) | 0.62 | 1.17 (0.54, 2.52) | 0.69 | 0.68 |
| + dietary patterns and BMI ^cd^ |  | 0.78 (0.30, 2.02) | 0.61 | 1.24 (0.55, 2.77) | 0.60 | 0.68 |
| Stratified analysis for BMI |  |  |  |  |  |  |
| BMI<23 kg/m^2^ | 232/2643 |  |  |  |  |  |
| Alcohol intake, %E (range) |  | (0.0–31.7) |  | - |  |  |
| Prevalence ratios (95% CI) |  |  |  |  |  |  |
| Adjusted for sex and other potential confounders ^b^ |  | 0.78 (0.48, 1.25) | 0.30 | 1.17 (0.98, 1.39) | 0.09 | 0.07 |
| + dietary patterns ^c^ |  | 0.64 (0.37, 1.13) | 0.12 | 1.19 (0.99, 1.43) | 0.06 | 0.10 |
| + BMI ^d^ |  | 0.70 (0.43, 1.12) | 0.13 | 1.18 (0.99, 1.41) | 0.06 | 0.10 |
| + dietary patterns and BMI ^cd^ |  | 0.65 (0.38, 1.10) | 0.11 | 1.18 (0.99, 1.41) | 0.06 | 0.11 |
| BMI≥23 kg/m^2^ | 844/1676 |  |  |  |  |  |
| Alcohol intake, %E (range) |  | (0.0–32.2) |  | - |  |  |
| Prevalence ratios (95% CI) |  |  |  |  |  |  |
| Adjusted for sex and other potential confounders ^b^ |  | 0.90 (0.75, 1.07) | 0.22 | 1.05 (0.98, 1.12) | 0.16 | 0.36 |
| + dietary patterns ^c^ |  | 0.85 (0.70, 1.04) | 0.12 | 1.06 (0.99, 1.13) | 0.09 | 0.27 |
| + BMI ^d^ |  | 0.93 (0.79, 1.10) | 0.39 | 1.03 (0.97, 1.1) | 0.31 | 0.53 |
| + dietary patterns and BMI ^cd^ |  | 0.89 (0.74, 1.08) | 0.25 | 1.04 (0.98, 1.11) | 0.23 | 0.45 |

^a^ Outliers for alcohol intake were excluded from the analysis, and separately defined according to sex as follows: more than the third quartile plus 1.5-times the interquartile range (≥32.26%E for men and ≥14.5%E for women).

^b^ Adjusted for age (years), smoking habit (current-smoker; ex-smoker; never smoker), and habitual exercise (almost never; 1–2 days/week; 3–5 days/week; everyday).

^c^ Additionally adjusted for the four factor scores for the four dietary patterns.

^d^ Additionally adjusted for linear and squared terms for BMI.

BMI, body mass index; 95% CI, 95% confidence interval; %E, % of energy.

**Table S6. Associations of different types of alcoholic beverages with fatty liver in 4,319 adults enrolled in St Luke’s International Hospital’s annual health check-up program in Japan. ^a^**

| Sources of alcohol (range in % energy) and model covariates | Linear term  (per 5% energy) | Squared term  (per 5% energy^2^) | P for likelihood ratio test |
| --- | --- | --- | --- |
| Sake (0.0% to 23.4%) |  |  |  |
| Adjusted for potential confounders and dietary patterns ^b^ | 1.18 (0.96,1.45) | 0.95 (0.88,1.04) | 0.19 |
| + BMI ^c^ | 1.13 (0.93,1.37) | 0.97 (0.89,1.05) | 0.35 |
| + other alcoholic beverages ^d^ | 1.13 (0.93,1.37) | 0.96 (0.89,1.05) | 0.37 |
| Beer (0.0% to 26.6%) |  |  |  |
| Adjusted for potential confounders and dietary patterns ^b^ | 0.79 (0.64,0.98) | 1.00 (0.92,1.09) | 0.001 |
| + BMI ^c^ | 0.90 (0.72,1.11) | 0.99 (0.90,1.08) | 0.05 |
| + other alcoholic beverages ^d^ | 0.91 (0.73,1.14) | 0.98 (0.90,1.08) | 0.07 |
| Shochu (0.0% to 30.8%) |  |  |  |
| Adjusted for potential confounders and dietary patterns ^b^ | 1.03 (0.90,1.18) | 1.00 (0.96,1.03) | 0.57 |
| + BMI ^c^ | 0.96 (0.85,1.09) | 1.01 (0.98,1.04) | 0.65 |
| + other alcoholic beverages ^d^ | 0.96 (0.85,1.09) | 1.01 (0.98,1.04) | 0.68 |
| Whisky (0.0% to 24.3%) |  |  |  |
| Adjusted for potential confounders and dietary patterns ^b^ | 1.18 (0.96,1.44) | 0.98 (0.92,1.05) | 0.05 |
| + BMI ^c^ | 1.05 (0.88,1.26) | 1.00 (0.94,1.07) | 0.26 |
| + other alcoholic beverages ^d^ | 1.04 (0.87,1.24) | 1.01 (0.95,1.07) | 0.35 |
| Wine (0.0% to 17.8%) |  |  |  |
| Adjusted for potential confounders and dietary patterns ^b^ | 0.85 (0.66,1.09) | 1.03 (0.91,1.16) | 0.10 |
| + BMI ^c^ | 0.87 (0.70,1.07) | 1.05 (0.95,1.15) | 0.32 |
| + other alcoholic beverages ^d^ | 0.87 (0.70,1.08) | 1.05 (0.95,1.16) | 0.35 |

^a^ n cases=1,076 and n total=4,319 after excluding outliers of alcohol consumption (more than the third quartile plus 1.5-times the interquartile range (≥32.26%E for men and ≥14.5%E for women)).

^b^ Adjusted for age (years), smoking habit (current-smoker; ex-smoker; never smoker), habitual exercise (almost never; 1–2 days/week; 3–5 days/week; everyday), four factor scores for the four dietary patterns.

^c^ Linear and squared terms of BMI

^d^ Five subtypes of alcoholic beverages were included in the same model.
